# Impact of Career Readiness Levels on Professional Identity in Nursing

**DOI:** 10.7759/cureus.102692

**Published:** 2026-01-31

**Authors:** Reiji Higashida, Ryoma Tanaka, Ryohei Miki, Miyuki Yamamoto

**Affiliations:** 1 Department of Nursing Science, School of Health Sciences, Faculty of Medicine, Hirosaki University, Hirosaki , JPN; 2 Department of Nursing Science, School of Health Sciences, Faculty of Medicine, Hirosaki University, Hirosaki, JPN; 3 Department of Nursing Science, Hirosaki University Graduate School of Health Sciences, Hirosaki, JPN

**Keywords:** career development, career readiness, life career perspectives, nursing students, occupational career planning, professional identity, social contribution

## Abstract

Introduction: In nursing education, career readiness and professional identity are regarded as key factors influencing students’ career development. Previous studies have suggested that their interaction contributes to effective career formation. Therefore, this study examined differences in professional identity between nursing students with high and low levels of career readiness.

Methods: This analytical cross-sectional questionnaire survey was conducted among 329 first- to fourth-year male and female nursing students enrolled in a four-year program at Hirosaki University in Hirosaki, Japan. The Career Readiness Scale-Short Version (CRS-S) and the Professional Identity Scale for Medical University Students were used. A total of 157 valid responses were analyzed (valid response rate: 47.7%). Participants were divided into high- and low-score groups for each subscale of the Career Readiness Scale, and differences in the four professional identity factors were compared between groups using Mann-Whitney U tests, and associations were examined using Spearman’s rank correlation coefficient.

Results: For Occupational Career Concern (OCC), students in the high career readiness group showed significantly higher median scores than those in the low group across all professional identity items, including confidence in choosing a healthcare profession (p < 0.001), establishment of a professional self-concept (p < 0.001), pride in being needed as a healthcare professional (p < 0.01), and orientation toward social contribution (p < 0.01). Overall, career readiness levels were positively correlated with all professional identity factors (p < 0.01). In the low Occupational Career Autonomy (OCA) group, positive correlations were observed for the establishment of a professional self-concept (rs = 0.733, p < 0.01) and confidence in choosing a healthcare profession (rs = 0.543, p < 0.05). Additionally, Life Career Planning (LCP) was positively correlated with the establishment of a professional self-concept (rs = 0.530, p < 0.01).

Conclusions: Nursing students with higher career readiness tended to exhibit a professional identity, particularly in terms of their sense of social contribution as future healthcare professionals. Among students with lower career readiness, associations with professional identity were limited. OCA was linked to pride in choosing the healthcare profession and to the formation of personal views on healthcare, whereas LCP was related only to the formation of personal views on healthcare. These findings suggest that differences in the level of career readiness promote the development of professional identity in nursing education.

## Introduction

In recent years, Japan has been experiencing rapid population aging and declining birth rates [1,2]. By 2030, it is estimated that more than 30% of the population will be aged 65 years or older, while nearly 20% will be aged 75 years or older; consequently, one in every three people in Japan will be a senior citizen [1]. In addition, the total fertility rate has remained well below the replacement level, and the number of live births has continued to decline over recent decades [2]. Although the number of employed nursing professionals has been steadily increasing [3], the demand for nurses remains high, and concerns over a nursing shortage persist. Nursing student enrollment has declined since fiscal year 2019 due to the effects of the declining birth rate [4]. Previous studies revealed that nursing students were more likely than students in other fields to have made early career decisions [5,6]. Many begin imagining themselves as nurses during elementary or junior high school and eventually enroll in nursing programs as initially hoped [6]. Furthermore, a relationship has been reported between nursing students’ intrinsic motivation, such as a desire to contribute to others or society, and a concern for academics, and subsequent career maturity [7]. In addition, as students advance through their academic years and gain practical training experience, their career image of the nursing profession becomes more concrete. At the same time, they also encounter the challenges and demands of the nursing field [8,9].

Career maturity is defined as “an individual’s readiness and attitude towards making career choices, decisions, and subsequent adaptation,” and within this concept, career readiness affects future outlooks [8]. In other words, nurturing a firm commitment to becoming a nurse and maintaining the motivation to continue one’s studies despite challenges [10] are considered important perspectives in supporting career development and maturity. On the other hand, professional identity is defined as “a sense of unity between the self and one’s profession” [11], and its establishment during the student years is considered to be strongly associated with career development and maturity [9].

In nursing education, career readiness and professional identity are regarded as important factors influencing students’ career development [10-18]. The Model Core Curriculum emphasizes the development of professional qualities and competencies necessary for future practice [10]. Previous studies related to the curriculum have reported efforts to strengthen professional identity among academically underperforming students [13], the importance of program continuity for better curricular integration and student support [14], and the recognition of professional competence as a key predictor of the hidden curriculum in nursing education [15]. Notably, the level of professional identity among nursing students has been shown to be closely associated with career planning [16]. In addition, studies have focused on prioritizing individuals with low confidence in clinical nursing practice among clinical instructors and administrators [9], assessing low levels of career maturity in nursing students [17], and examining the formation of professional identity among male nurses [18]. Collectively, these studies highlight a strong research interest in professional identity and levels of career maturity. Educational approaches that effectively integrate career readiness and professional identity may therefore contribute to a more meaningful undergraduate nursing program and influence subsequent career development.

Although previous studies reported on the relationship between career maturity and motivation for career choice among nursing university students [7], and another study addressed the relationship between career readiness and vocational career maturity in nursing students [19], the relationship between professional identity and career readiness across different levels of career readiness among nursing students has yet to be investigated.

Therefore, the aim of this study was to examine differences in professional identity between nursing students with high and low levels of career readiness, as well as the associations between career readiness and professional identity. Clarifying the association between career maturity and professional identity may help nursing students better understand the meaning of career development and enhance their preparedness through enriched career education. At the same time, such findings may assist educators in creating more supportive academic environments. This study, therefore, aims to provide foundational evidence for the development of effective career education in nursing programs.

## Materials and methods

Subjects and study period

This study was an analytical observational study. Participants were recruited using convenience sampling based on a non-probability (purposive) sampling method.

Subjects were 329 male and female nursing students from the first to fourth years of the four-year nursing program at Hirosaki University in Hirosaki, Japan. The survey was conducted between April 5 and April 30, 2024. Only students who expressed willingness to participate and provided informed consent were allowed to complete the questionnaire. Assuming a response rate of 45% and a margin of error of 5.7%, a required sample size of 300 participants was calculated. To account for potential absentees on the survey day and missing data, questionnaires were distributed to a total of 329 students.

Inclusion criteria were (1) enrollment as a first- to fourth-year student in the nursing program at Hirosaki University, (2) consent to participate in the study, and (3) completion of the questionnaire. Exclusion criteria were (1) non-consent to participation and (2) incomplete responses. All respondents completed every questionnaire item; therefore, no participants were excluded due to missing data.

Study methods

Data were collected through a web-based questionnaire created using Microsoft Forms (Microsoft Corp., Redmond, WA, USA). The questionnaire in Japanese with its English translation is provided in Appendix A. The purpose of the study and the request for participation were explained orally to second- to fourth-year students following a guidance session held at the beginning of the 2024 academic year. The explanation of the study was provided orally to first-year students after the first lecture of the general education course “Introduction to Data Science,” with the cooperation of the faculty member in charge of the course (from the Department of Nursing). Participants were informed in advance that the survey would take approximately five minutes to complete.

Study contents

The questionnaire consisted of the following contents: (1) subject characteristics, including academic year, sex, and age; (2) Career Readiness Scale-Short Version (CRS-S); and (3) the Professional Identity Scale.

Career Readiness Scale-Short Version

The CRS-S, developed by Sakayanagi [8], was used in this study. The CRS-S consists of two domains: Occupational Career Readiness (OCR) and Life Career Readiness (LCR), each of which is further divided into three subdomains. OCR includes Occupational Career Concern (OCC), Occupational Career Autonomy (OCA), and Occupational Career Planning (OCP), whereas LCR includes Life Career Concern (LCC), Life Career Autonomy (LCA), and Life Career Planning (LCP).

Participants responded to items assessing each subdomain. For OCC, they indicated the extent to which they were interested in their future occupation and work style, sought information to enrich their working life, and considered their future work to be an important issue. For OCA, they rated the degree to which they intended to shape their working life through their own efforts, planned how to spend their working life independently, and actively challenged themselves to enhance their occupational experience. For OCP, participants evaluated whether they had concrete plans to achieve their desired working style, held personal goals regarding the type of professional they wanted to become, and engaged in planned actions to lead a fulfilling working life.

Similarly, for the LCR subdomains, participants rated their interest in their future life and lifestyle, their efforts to access information to enrich life, and the seriousness with which they considered how to live in the future for LCC. For LCA, they indicated the extent to which they intended to shape their life through their own efforts, independently considered how to live, and actively challenged themselves to lead a fulfilling life. For LCP, participants reported whether they had concrete plans to achieve their desired lifestyle, held personal goals regarding the kind of person they wanted to become, and engaged in planned actions to lead a fulfilling life.

Each domain of the CRS-S consists of three subdomains-career concern, career autonomy, and career planning-with three items per subdomain, yielding a total of 18 items across the scale. Responses were rated using a five-point Likert scale: “5 = Strongly applicable,” “4 = Somewhat applicable,” “3 = Neutral,” “2 = Slightly inapplicable,” and “1 = Not applicable at all”. Each subdomain score ranged from 3 to 15, with higher scores indicating greater career readiness (i.e., a higher level of career maturity) in the respective domain. In the analysis, subdomain scores of ≤9 were categorized as the low-score group, whereas scores of ≥10 were categorized as the high-score group.

Internal consistency reliability of the CRS-S in the present study was acceptable to good, with Cronbach’s alpha coefficients of 0.79 for OCC, 0.73 for OCA, 0.76 for OCP, 0.83 for LCC, 0.83 for LCA, and 0.85 for LCP. 

Professional Identity Scale for medical university students

The Professional Identity Scale for medical university students, developed by Fujii et al. [20], was used in this study. Factor analysis of the original 34 items yielded four factors, from which the five items with the highest factor loadings for each factor were selected, resulting in a total of 20 items.

Based on this, Ochiai et al. [6] further shortened the scale by selecting the top five items for each of the four factors (F1, F2, F3, and F4), resulting in a 20-item version with five items per factor, which was used as a reference in the present study. The scale consists of the following four factors [6, 20].

The Four Factors

The four factors are as follows: F1: Confidence in choosing a healthcare profession, F2: Establishment of one’s own professional view of healthcare work, F3: Pride in being needed as a healthcare professional, and F4: Orientation towards social contribution.

For F1: participants responded to items such as whether they thought choosing nursing was a good decision, whether they could not imagine working in any other occupation, whether they intended to continue nursing throughout their lives, whether they felt that becoming a nurse was an authentic way of life for them, and whether they could proudly state to others that they were nursing students.

For F2: tems assessed whether participants had a clear idea of the kind of nursing they wanted to practice, whether they felt capable of providing nursing care that reflected their individuality, whether they had a clear vision of the kind of nurse they wanted to become, whether they believed they would be able to practice nursing in their own way in the future, and whether they felt they had their own ideas regarding the nature of nursing.

For F3: With the introductory statement “As a nurse…,” participants were asked whether they believed they were an indispensable presence in the healthcare field, whether they had been and would continue to be needed by many people, whether they would increasingly be required as members of healthcare teams, and whether they believed nursing was grounded in its own academic discipline.

For F4: Participants responded to items regarding whether they wished to contribute to patients, to meet patients’ needs, to contribute to society, to advance medical care, and to further the development of nursing as a discipline.

Responses were rated on a seven-point Likert scale, from “7 = Strongly applicable” to “1 = Not applicable at all,” and the average total score was calculated. For each factor, the minimum possible score was 5, and the maximum possible score was 35. Higher scores indicated a higher level of professional identity. Scores ranged from 1 to 7, with higher scores indicating a stronger level of professional identity. Internal consistency reliability was high, with Cronbach’s alpha coefficients of 0.91 for Factor 1 (F1), 0.87 for Factor 2 (F2), 0.95 for Factor 3 (F3), and 0.96 for Factor 4 (F4).

Data analysis

Descriptive statistics (frequencies, percentages, means, and standard deviations) were used to summarize participant characteristics. The scores from the CRS-S and the Professional Identity Scale for Medical University Students were analyzed using the Mann-Whitney U test, as the data did not meet the assumptions of normal distribution. Furthermore, the relationship between career readiness and professional identity was examined using Spearman's rank correlation coefficient. All statistical analyses were conducted using IBM SPSS Statistics, version 25 (IBM Corp., Armonk, NY, USA). A p-value of less than 0.05 was considered statistically significant.

Ethical considerations

Subjects were informed of the purpose of the study, that participation was voluntary, and that their privacy would be protected with no personally identifiable information collected. The same information was also provided in written form via the web-based questionnaire and in the e-mail used to distribute it. At the beginning of the questionnaire in Microsoft Forms, participants were asked to indicate their consent by selecting “Agree” or “Disagree”. Consent was considered to be obtained when “Agree” was selected. The survey was anonymized by disabling name recording in the form settings. After collecting responses, the data were exported to Microsoft Excel (Microsoft Corp.), and the distribution history (Office 365 Forms, Microsoft Corp.) was deleted. The form settings allowed only one response per subject.

This study was conducted with the approval of the Graduation Research Ethics Review Committee of the School of Health Sciences, Faculty of Medicine, Hirosaki University (approval no.: HS2023-080).

## Results

Subject characteristics

Among 329 nursing students, 157 responses were valid (valid response rate: 47.7%). First-year students accounted for the largest group with 56 students (35.7%), followed by fourth-year students with 49 students (31.2%). There were 19 male students (12.1%) and 138 female students (87.9%). The overall mean age was 19.5 ± 1.3 years (mean ± standard deviation) (Table 1).

**Table 1:** Basic attributes of the study group (n=157) *SD: standard deviation

Item		Number of students	Percentage (%; or SD)
Academic year	First-year students	56	35.7
	Second-year students	26	16.6
	Third-year students	26	16.6
	Fourth-year students	49	31.2
Sex	Male	19	12.1
	Female	138	87.9
Mean age (years)	All	19.5	±1.3*
	Male	19.6	±1.5*
	Female	19.5	±1.5*

Relationship between OCR and professional identity among medical university students (group comparison)

For the CRS-S, subdomain scores ranged from 3 to 15. Based on predefined criteria, participants with scores of ≤9 were categorized into the low-score group, whereas those with scores of ≥10 were categorized into the high-score group. These groupings were applied consistently across analyses to examine differences in professional identity factors.

Regarding OCC, the median scores of the high-score group were significantly higher than those of the low-score group for all four professional identity factors: F1 (p<0.001), F2 (p<0.001), F3 (p<0.01), and F4 (p<0.01). Concerning OCA, the high-score group scored significantly higher than the low-score group on F1 (p<0.001) and F4 (p<0.05), whereas no significant difference was observed for F3. For F2, although the median scores were identical between the two groups, the interquartile range (IQR) was significantly higher in the low-score group (IQR: 22.0-31.0).

With regards to OCP, the high-score group scored significantly higher than the low-score group on F3 (p<0.01) and F4 (p<0.01). In contrast, the median scores for F1 and F2 were identical between the two groups. However, for F1, the IQR was significantly higher in the low-score group (IQR: 20.0-30.0), whereas for F2, the IQR was significantly higher in the high-score group (IQR: 18.9-30.0) (Table 2).

**Table 2:** Relationship between occupational Career Readiness Scales-Short Version and professional identity among medical university students (n=157) Values are presented as medians and interquartile ranges. p-values were calculated using the Mann–Whitney U test. n: number of students; F1: Confidence in choosing a healthcare profession; F2: Establishment of one’s own professional view of healthcare work; F3: Pride in being needed as a healthcare professional; F4: Orientation toward social contribution

Subscale items		High-score group (Occupational Career Readiness)			Low-score group (Occupational Career Readiness)			p
		n	Median	25^th^–75^th^ percentiles	n	Median	25^th^–75^th^ percentiles	
Occupational Career Concern	F1	139	26.0	20.0-30.0	18	18.5	14.0-22.3	p<0.001
	F2		25.0	21.0-30.0		13.0	8.3-20.3	p<0.001
	F3		25.0	20.0-31.0		18.0	9.8-23.5	p<0.01
	F4		32.0	28.0-35.0		27.0	20.5-31.0	p<0.01
Occupational Career Autonomy	F1	142	25.0	20.0-30.0	15	24.0	20.0-29.0	p<0.001
	F2		25.0	18.0-30.0		25.0	22.0-31.0	p<0.001
	F3		24.0	20.0-31.0		25.0	20.0-29.0	0.134
	F4		31.5	27.0-35.0		30.0	25.0-35.0	p<0.05
Occupational Career Planning	F1	106	25.0	19.8-30.0	51	25.0	20.0-30.0	p<0.001
	F2		25.0	18.9-30.0		25.0	19.0-29.0	p<0.001
	F3		25.0	20.0-32.3		23.0	20.0-27.0	p<0.01
	F4		31.5	28.0-35.0		31.0	25.0-35.0	p<0.01

Relationship between LCR and professional identity among medical university students (group comparison)

Regarding LCC, the high-score group had significantly higher median scores than the low-score group for F1 (p<0.01) and F4 (p<0.01). For F2, although the median scores were identical between the two groups, the IQR was significantly higher in the low-score group (IQR: 21.5-32.5; p < 0.001). In addition, the low-score group scored significantly higher than the high-score group on F3 (p < 0.05).

For F2, the median score was also significantly higher in the high-score group (p < 0.05), whereas for F3, the low-score group showed a significantly higher median score than the high-score group (p < 0.001). Concerning LCA, the high-score group had significantly higher scores for F1 (p<0.001) and F4 (p<0.05). In contrast, for F2, the median score was significantly higher in the low-score group (p < 0.001), and no significant difference was observed for F3. Regarding LCP, the high-score group had significantly higher median scores than the low-score group for F3 (p < 0.01) and F4 (p < 0.01). Although the median scores for F1 and F2 were identical between the two groups, the IQRs were significantly higher in the high-score group for F1 (IQR: 20.0-30.0) and F2 (IQR: 20.0-30.0) (Table 3).

**Table 3:** Relationship between life Career Readiness Scales-Short Version and professional identity among medical university students (n=157) Values are presented as medians and interquartile ranges. p-values were calculated using the Mann–Whitney U test. n: number of students; F1: Confidence in choosing a healthcare profession; F2: Establishment of one’s own professional view of healthcare work; F3: Pride in being needed as a healthcare professional; F4: Orientation toward social contribution Establishment of one’s own professional view of healthcare work

Subscale items		High-score group (Life Career Readiness)			Low-score group (Life Career Readiness)			p
		n	Median	25^th^–75^th^ percentiles	n	Median	25^th^–75^th^ percentiles	
Life Career Concern	F1	144	25.0	20.0-30.0	13	23.0	19.5-30.0	p<0.01
	F2		25.0	19.0-30.0		25.0	21.5-32.5	p<0.001
	F3		24.0	20.0-30.5		25.0	18.5-29.0	p<0.05
	F4		31.0	27.0-35.0		30.0	25.0-35.0	p<0.01
Life Career Autonomy	F1	145	25.0	20.0-30.0	12	22.0	19.3-30.5	p<0.001
	F2		25.0	18.5-30.0		26.5	21.8-33.3	p<0.001
	F3		24.0	20.0-30.5		25.0	17.8-30.5	0.130
	F4		32.0	27.0-35.0		30.0	25.0-34.8	p<0.05
Life Career Planning	F1	116	25.0	20.0-30.0	41	25.0	20.0-29.5	p<0.001
	F2		25.0	20.0-30.0		25.0	17.5-30.0	p<0.001
	F3		25.0	20.0-32.0		23.0	18.0-26.5	p<0.01
	F4		32.0	28.0-35.0		31.0	25.0-35.0	p<0.01

Relationship between levels of career readiness and professional identity (correlation analysis)

In all high career readiness groups, overall weak to moderate positive correlations were observed between each dimension of career readiness and professional identity across all factors (p < 0.01). In the low OCA group, a strong positive correlation was found between professional identity F2, representing the establishment of personal views on the healthcare profession, and OCA (Spearman’s rs = 0.733, p < 0.01). In addition, the low OCA group showed a positive correlation between professional identity F1, reflecting confidence in career choice, and OCA (rs = 0.543, p < 0.05). Furthermore, in the low LCC group, a positive correlation was observed between professional identity F2 (establishment of personal views on the healthcare profession) and LCC (rs = 0.530, p < 0.01) (Table 4).

**Table 4:** Correlations between levels of career readiness and professional identity ** Correlation coefficients are significant at the 1% level (two-tailed); * Correlation coefficients are significant at the 5% level (two-tailed); Correlation coefficients were calculated using Spearman’s rank correlation.

			Occupational Career Concern	Occupational Career Autonomy	Occupational Career Planning	Life Career Concern	Life Career Autonomy	Life Career Planning
			High-score group n=139	High-score group n=142	High-score group n=106	High-score group n=144	High-score group n=145	High-score group n=116
			Low-score group n=18	Low-score group n=15	Low-score group n=51	Low-score group n=13	Low-score group n=12	Low-score group n=41
			Correlation coefficients	Correlation coefficients	Correlation coefficients	Correlation coefficients	Correlation coefficients	Correlation coefficients
Professional Identity Scale for medical university students	F1: Confidence in choosing a healthcare profession	High-score group	0.327^**^	0.336^**^	0.499^**^	0.456^**^	0.459**	0.495**
	F2: Establishment of one’s own professional view of healthcare work		0.369^**^	0.458^**^	0.545^**^	0.481^**^	0.511**	0.451**
	F3: Pride in being needed as a healthcare professional		0.229^**^	0.234^**^	0.287^**^	0.385^**^	0.309**	0.326**
	F4: Orientation towards social contribution		0.350^**^	0.396^**^	0.405^**^	0.440^**^	0.470**	0.369**
	F1: Confidence in choosing a healthcare profession	Low-score group	0.214	0.543^*^	0.159	0.236	0.227	0.239
	F2: Establishment of one’s own professional view of healthcare work		-0.108	0.733^**^	0.298^**^	0.218	0.33	0.530**
	F3: Pride in being needed as a healthcare professional		0.109	0.431	0.227	-0.054	-0.047	0.126
	F4: Orientation towards social contribution		0.144	0.234	0.127	0.193	0.172	0.119

## Discussion

This study examined the relationship between OCR and LCR with professional identity. In Japan, various governmental policies have been implemented to promote work-life balance and the creation of a gender-equal society, aiming to enrich the quality of social life for its citizens. Within this context, students are engaged in the formation of their professional identity as nurses [20]. In this study, significant correlations were observed between career readiness and all dimensions of professional identity in the high career readiness group. This finding is consistent with the notion that professional identity develops through recognition of oneself as a member of a specific group [21], suggesting that students majoring in nursing may internalize such awareness through their daily academic and clinical experiences. In contrast, in the LCR group, professional identity F2, which represents the establishment of independent personal views on the healthcare profession, showed a higher median score and a strong positive correlation. This suggests that these students may consciously choose to major in nursing while recognizing this aspect of professional identity as a minimum requirement for psychological growth, integrity, and well-being [22].

OCC consists of keywords such as interest, information, and commitment [8]. In the present study, students in the high-score group showed higher median scores than those in the low-score group across all four factors of the Professional Identity Scale: F1 “Confidence in choosing a healthcare profession,” F2 “Establishing one’s role as a healthcare professional,” F3 “Pride in being needed as a healthcare professional” and F4 “Orientation toward social contribution.” In particular, with regard to F1, students appear to have clear goals from the time of admission, such as obtaining professional qualifications and developing specialized expertise [23], which may contribute to a sense of integration between the self and the profession as part of their professional identity. The participants were already interested in the nursing profession, and the results indicated that they held a strong awareness of their professional identity.

Previous reports have noted that attitudes, values, and beliefs function as readiness for behavior [24]. Similarly, in this study, a higher level of OCC was associated with a stronger professional identity. Given that professional identity has also been reported to influence profession-specific behaviors such as caring [25], the findings suggest that, within nursing education, students with higher OCC are more motivated to engage in their studies with a strong sense of professional identity. In contrast, LCC did not show such a pronounced tendency in relation to professional identity as OCC did, even among students in the high-score group. Previous studies have suggested that the formation of professional identity requires practical training experiences and the meaning attributed to those experiences [21], which may explain why students are able to conceptualize nursing primarily as a “profession.” However, with regard to exercising one’s abilities (competence), feeling connected to others (relatedness), and making self-directed and coherent choices (autonomy) [22], it can be inferred that students may not yet have reached a stage at which these elements are fully integrated into their broader life course and personal growth.

Furthermore, as shown in Table 3, for F2, the high-score group scored significantly lower in LCC. Table 2 also shows that the majority of subjects in this study were female. Previous studies reported that nursing students were often preoccupied with exams and job-hunting [7] and that women, in particular, generally bear significant responsibilities within the household [26]. These findings suggest that, in relation to F2 (OCC), students may be too focused on immediate tasks, such as exams and employment, to envision their future lives or to imagine themselves working as nurses in the long term. Particularly with regard to OCC, occupational identity showed a significant difference in the median scores between the high- and low-score groups, with the high group scoring higher. However, this trend was not observed for LCR. This may be due to factors such as having chosen nursing as a major unconsciously or having limited confidence in their ability to engage in clinical nursing practice [9]. Since narrowing the target group has been shown to help underserved adolescents realize their aspirations for higher education and future careers [27], it is necessary to provide pre-graduation guidance tailored to each individual.

No significant differences were observed in OCA or LCA in relation to F3. A previous study demonstrated that students who had not yet entered clinical practice often found it difficult to develop a sense of pride in being needed as healthcare professionals [6]. Although the present study targeted only nursing students, similar results were observed. This suggests that not only limited experiences, such as clinical training, but also broader real-world clinical experiences as working professionals are important in fostering this aspect.

Since only a small number of students participate in career guidance sessions and webinars [28], previous research has reported the need for intervention programs aimed at improving emotional management abilities and emotional application skills [9]. Furthermore, in university education programs, it is necessary to increase training opportunities, such as role-playing, to enhance students’ “sense of pride in being needed as healthcare professionals” [6]. In addition, similar to structured career development initiatives implemented during doctoral training [29], it is also essential to provide students with guidance that conveys the availability of postgraduate education opportunities.

In a study by Ochiai et al. [6] that compared the Professional Identity Scale for medical university students and the types of career decision-making processes, students showed a strong orientation towards social contribution. Similarly, the present results demonstrated that OCR and LCR were both associated with a high orientation towards social contribution (F4), supporting previous findings. These results suggest that students studying healthcare generally possess a strong desire to contribute to society. According to Table 3, the high-score group had significantly higher scores for F4 of professional identity than the low-score group. Additionally, Table 3 shows that many students cited reasons such as “being able to support patients and their families up close” and “wanting to be helpful to others” as their motivation for choosing a nursing major. These results are consistent with previous findings [6]. Therefore, students in the high career readiness group view nursing as a profession fundamentally rooted in contributing to people and society, and nursing students generally have a strong orientation towards people and social contribution. To promote subjective well-being and help students approach new challenges in social contexts, individual and group interventions should be proposed [30]. Doing so can serve as motivation for enhancing their sense of well-being.

This study has several limitations. First, it was conducted at a single university and included only nursing majors, which restricts the generalizability of the findings. Second, although participants were enrolled in different tracks-nursing, public health nursing, midwifery, and teaching-they were uniformly treated as “nursing professions” with respect to career readiness. Given the distinct career paths involved (e.g., hospital nurses, public health nurses working under Japan’s unique lifelong employment system, and other trajectories), evaluating them as a single group is inherently problematic. Therefore, future studies should divide participants into multiple groups and examine these differences separately. Third, imbalances in academic year and sex were present in the sample, and clinical training experience also varied by year. As students progress, they participate not only in clinical placements at various facilities but also in seminars and employment-support programs; therefore, both career readiness and professional identity may differ by academic year. Future studies should examine these aspects by grade level. Finally, because this was a cross-sectional study, research involving larger populations and longitudinal designs is warranted.

## Conclusions

This study demonstrated that higher levels of career readiness among nursing students were associated with a stronger professional identity, particularly in terms of orientation toward social contribution. In particular, OCC showed a significant relationship with all factors of professional identity, indicating that interest, information-seeking, and commitment to one’s career play a central role in shaping the identity of future nurses. In contrast, LCC was less strongly related to professional identity, suggesting that students tend to prioritize immediate occupational aspects, such as exams and employment, over long-term life career perspectives.

These findings highlight the importance of fostering career readiness, especially OCC, within nursing education as a means of strengthening professional identity. To achieve this, structured educational interventions, such as role-playing, career guidance, and opportunities for reflection, should be integrated into the curriculum. Furthermore, tailored support for students to balance occupational and life career perspectives may help promote a more comprehensive and sustainable professional identity. Future longitudinal studies involving diverse nursing programs are warranted to clarify how career readiness contributes to identity formation throughout academic and professional development.

## Appendices

Appendix A

Questionnaire in Japanese

質問票（日本語）

看護学生におけるキャリアレディネスレベルが職業的アイデンティティに受ける影響

近年、我が国では少子高齢化が進行しています。2030年には日本の人口の3人に1人が高齢者になる見込みであるといわれていますが、依然として看護職員の需要は高く、看護師不足が懸念されています。先行研究によると看護学科では幼いころから看護師になることをイメージし、ほぼ希望通りに進学している人が多いといわれています。また、看護職種の志望動機が社会への貢献や学問への興味といった内発的動機とその後のキャリア形成に影響していると述べられています。

キャリア成熟とは、「キャリアの選択・決定やその後の適応への個人のレディネス、取り組み姿勢」と定義されているものであり、この中のキャリアレディネスが将来像に実際に影響しているといわれています。つまり、看護師になると言う決意を育み、困難があっても学業を続けていく姿勢を保つことが、キャリア形成・成熟への支援に重要な視点であると考えます。

職業的アイデンティティは「職業との自己一体感」と定義されており、学生時代から確立されることで、キャリア形成・成熟に強い関連があるのではないかと考えます。先行研究では、看護学生におけるキャリア成熟度と職業選択志望動機との関連の報告や看護大学生のキャリアレディネスと職業的キャリア成熟の関連を報告した論文はありますが、キャリアレディネスレベルと職業的アイデンティティとの関連についての報告はありません。

そこで本研究では、看護学生のキャリアレディネスレベルが職業的アイデンティティにおいてどのように影響しているのかを検討することを目的としています。

この研究は5分ほどお時間がかかりますが、参加のための費用負担はありません。また、参加は自由意志であり、回答を拒否することもできます。同意拒否による不利益を与えることはなく、本調査は個人が特定されないように匿名化します。なお、一旦同意後の参加拒否は匿名化しているためできないことご了承ください。本調査の集計結果は、研究のみ使用し、成果物として発表いたします。

アンケートの集計データは指導教員である弘前大学保健学研究科看護学領域講師、山本美由紀研究室でメモリ媒体にして、厳重に10年間保管します。10年後はメモリ媒体の本研究データはすべて破棄します。ご意見お問い合わせは、下記の連絡先までお願いします。なお、本研究は弘前大学医学部保健学科卒業研究倫理委員会の承認を得て実施しています。

ご多忙のところ恐縮ですが、以上の研究趣旨をご理解いただき、ご協力いただきますようお願い申し上げます。

2024年4月30日までにご回答よろしくお願いいたします。

弘前大学医学部保健学科看護学専攻　東田怜二、田中稜真、三木涼平

弘前大学大学院保健学研究科講師　指導教員　山本美由紀

　TEL:0172-39-5943   E-mail:miyukiya@hirosaki-u.ac.jp

質問事項

１． 私は本課題について上記の説明をよく読み、理解した上で協力することに同意します。

　〇同意する    〇同意しない

２． 学年

     〇1年生    〇2年生      〇3年生    〇4年生

３． あなたの年齢を教えてください（入力してください）。

      (     )歳

４． 性別

       〇男性    〇女性    〇その他

5.    以下の質問で最も当てはまるものを一つ選んでください。（丸を付けてください）

**Table 5:** 質問5. 以下の質問で最も当てはまるものを一つ選んでください。 （丸を付けてください）

		全くあてはまらない	あまりあてはまらない	どちらともいえない	ややあてはまる	よくあてはまる
1	これからの職業生活や働き方について、とても興味をもっている	１	２	３	４	５
2	職業生活を充実させるために、より多くの情報に接するようにしている	１	２	３	４	５
3	これからの働き方は自分にとって重要な問題なので、本気で考えている	１	２	３	４	５
4	これからの職業生活は、自分の力で切り開いていこうと思う	１	２	３	４	５
5	職業生活をどう過ごすかは、他人から言われなくても考えている	１	２	３	４	５
6	職業生活を充実させるためには、多くのことをチャレンジしようと思う	１	２	３	４	５
7	希望する働き方をするための具体的な計画を立てている	１	２	３	４	５
8	将来どのような職業人になりたいか、自分なりの目標を持っている	１	２	３	４	５
9	充実した職業生活を送るために、計画的に取り組んでいることがある。	１	２	３	４	５

6.    以下の質問で最も当てはまるものを一つ選んでください。（丸を付けてください）

**Table 6:** 質問6. 以下の質問で最も当てはまるものを一つ選んでください。 （丸を付けてください）

		全くあてはまらない	あまりあてはまらない	どちらともいえない	ややあてはまる	よくあてはまる
1	これからの人生や生き方について、とても興味をもっている	１	２	３	４	５
2	人生を充実させるために、より多くの情報に接するようにしている	１	２	３	４	５
3	これからの生き方は自分にとって重要な問題なので、本気で考えている	１	２	３	４	５
4	これからの人生は、自分の力で切り開いていこうと思う	１	２	３	４	５
5	人生をどう過ごすかは、他人から言われなくても考えている	１	２	３	４	５
6	人生を充実させるためには、多くのことをチャレンジしようと思う	１	２	３	４	５
7	希望する生き方をするための具体的な計画を立てている	１	２	３	４	５
8	将来どのような人になりたいか、自分なりの目標を持っている	１	２	３	４	５
9	充実した人生を送るために、計画的に取り組んでいることがある。	１	２	３	４	５

7.次の質問で最も当てはまるものを選んでください。（丸を付けてください）

**Table 7:** 質問7. 次の質問で最も当てはまるものを選んでください。 （丸を付けてください）

		全く当てはまらない	ほとんど当てはまらない	どちらかといえば当てはまらない	どちらでもない	どちらかといえば当てはまる	ほとんどあてはまる	非常にあてはまる
1	私は看護職を選択したことはよかったと思っている	１	２	３	４	５	６	７
2	私は看護職以外の仕事は考えられないと思っている	１	２	３	４	５	６	７
3	私は看護職を生涯続けようと思っている	１	２	３	４	５	６	７
4	私は看護職に就くことが自分らしい生き方だと思っている	１	２	３	４	５	６	７
5	私は看護職を志す学生であると、他人に誇りをもって言うことができると思う	１	２	３	４	５	６	７
6	自分がどんな看護をしたいかははっきりしていると思っている	１	２	３	４	５	６	７
7	私は自分らしい看護をしていくことができると思っている	１	２	３	４	５	６	７
8	自分がどんな看護師なりたいか、はっきりしていると思う	１	２	３	４	５	６	７
9	将来、自分らしい看護ができるようになると思う	１	２	３	４	５	６	７
10	私は看護のあり方について、自分なりの考えを持っていると思う	１	２	３	４	５	６	７
11	私は看護師として、医療の世界で不可欠な存在だと思っている	１	２	３	４	５	６	７
12	私は看護師として、これまでも、これからも、多くの人に必要とされていると思う	１	２	３	４	５	６	７
13	自分は看護師として患者に必要とされていると思う	１	２	３	４	５	６	７
14	私は看護師として、医療チームの一員として今後ますます必要とされると思う	１	２	３	４	５	６	７
15	私は看護師として、背景に独自の学問体系を持っていると思う	１	２	３	４	５	６	７
16	私は看護師として、患者に貢献していきたいと思っている	１	２	３	４	５	６	７
17	私は看護師として、患者の願いにこたえたいと思っている	１	２	３	４	５	６	７
18	私は看護師として、社会に貢献していきたいと思っている	１	２	３	４	５	６	７
19	私は看護師として、医療の発展に貢献したいと思っている	１	２	３	４	５	６	７
20	私は看護師として、看護の世界の発展に貢献していきたいと思っている	１	２	３	４	５	６	７

これで質問は以上です。ありがとうございました。

English Translation of the Questionnaire

The Impact of Career Readiness Levels on Professional Identity Among Nursing Students

In recent years, Japan has been experiencing rapid population aging accompanied by a declining birthrate. It is estimated that by 2030, one in three people in Japan will be elderly. Despite this demographic shift, the demand for nursing professionals remains high, and a shortage of nurses continues to be a major concern. Previous studies have reported that many students in nursing programs have envisioned becoming nurses from an early age and have enrolled in nursing schools largely in accordance with their original career aspirations. In addition, it has been suggested that intrinsic motivations for choosing the nursing profession, such as a desire to contribute to society or an interest in academic learning, have an influence on subsequent career development.

Career maturity is defined as “an individual’s readiness and engagement in career choice, decision-making, and subsequent adaptation.” In this framework, career readiness plays a particularly important role in shaping an individual’s future career image. Specifically, developing a strong commitment to becoming a nurse and sustaining motivation to continue academic studies despite difficulties are critical perspectives for supporting career formation and career maturity.

Occupational identity is defined as “a sense of unity between oneself and one’s profession,” and establishing this identity during the student years has been shown to be strongly associated with career development and career maturity. Previous studies have reported associations between career maturity and motivation for occupational choice among nursing students, as well as relationships between career readiness and occupational career maturity among undergraduate nursing students. However, to the best of our knowledge, there have been no reports examining the relationship between career readiness levels and occupational identity.

Thus, this study aims to examine how career readiness levels among nursing students influence their occupational identity, using a questionnaire survey.

Participation in this study will take approximately five minutes, and there is no financial cost associated with participation. Participation is entirely voluntary, and you may refuse to answer the questionnaire. There will be no disadvantages or penalties for refusing to participate. All responses will be anonymized so that individuals cannot be identified. Please note that once you have provided consent and submitted your responses, withdrawal from the study will not be possible due to the anonymous nature of the data. The aggregated results of this survey will be used solely for research purposes and will be reported for academic purposes.

The collected questionnaire data will be stored on a memory device and securely retained for ten years in the laboratory of the supervising faculty member, Ms. Miyuki Yamamoto, Lecturer of the Department of Nursing Science, Graduate School of Health Sciences, Hirosaki University. After ten years, all research data stored on the memory device will be permanently destroyed. If you have any questions or concerns regarding this study, please contact the Supervisor, Miyuki Yamamoto, at the contact information provided below. This study has been approved by the Graduation Research Ethics Committee of the Faculty of Health Sciences, Hirosaki University.

We sincerely appreciate your understanding of the purpose of this study and your cooperation despite your busy schedule. Please complete the questionnaire by April 30, 2024.

Investigators: Reiji Higashida, Ryoma Tanaka, Ryohei Miki

Department of Nursing Science, School of Health Sciences, Faculty of Medicine, Hirosaki University

Supervisor: Miyuki Yamamoto

Department of Nursing Science, Graduate School of Health Sciences, Faculty of Medicine, Hirosaki University　

TEL:0172-39-5943   E-mail:miyukiya@hirosaki-u.ac.jp

Questions

1. Consent to Participate I have carefully read and understood the explanation above regarding this study, and I agree to participate.

☐ I agree　　　☐ I do not agree

2. Academic Year Please select your current academic year.

☐ First year　　☐ Second year　　☐ Third year　　☐ Fourth year

3. Age Please enter your age.　　(         ) years

4. Sex

☐ Male　　☐ Female　　☐ Other

5. Please indicate how well each statement applies to you. (Please circle one response for each item)

**Table 8:** Quetion 5. Please indicate how well each statement applies to you. (Please circle one response for each item)

		Strongly disagree	Disagree	Neither agree nor disagree	Agree	Strongly agree
1	I am very interested in my future career and working life.	1	2	3	4	5
2	In order to lead a fulfilling career, I actively seek out a wide range of information.	1	2	3	4	5
3	My future way of working is an important issue for me, and I think seriously about it.	1	2	3	4	5
4	I intend to shape my future career through my own efforts.	1	2	3	4	5
5	I think about how I want to spend my working life without being told by others.	1	2	3	4	5
6	In order to have a fulfilling career, I am willing to take on many challenges.	1	2	3	4	5
7	I have made concrete plans to achieve the way of working that I hope for.	1	2	3	4	5
8	I have my own goals regarding what kind of professional I want to become in the future.	1	2	3	4	5
9	I am taking planned actions to achieve a fulfilling working life.	1	2	3	4	5

6. Please indicate how well each statement applies to you. (Please select one response for each item)

**Table 9:** Question 6. Please indicate how well each statement applies to you. (Please select one response for each item)

		Strongly disagree	Disagree	Neither agree nor disagree	Agree	Strongly agree
1	I am very interested in my future life and way of living.	1	2	3	4	5
2	In order to lead a fulfilling life, I actively seek out a wide range of information.	1	2	3	4	5
3	My future way of living is an important issue for me, and I think seriously about it.	1	2	3	4	5
4	I intend to shape my future life through my own efforts.	1	2	3	4	5
5	I think about how I want to live my life without being told by others.	1	2	3	4	5
6	In order to lead a fulfilling life, I am willing to take on many challenges.	1	2	3	4	5
7	I have made concrete plans to achieve the way of life that I hope for.	1	2	3	4	5
8	I have my own goals regarding what kind of person I want to become in the future.	1	2	3	4	5
9	I am taking planned actions to achieve a fulfilling life	1	2	3	4	5

7. Please indicate how well each statement applies to you. (Please select one response for each item)

**Table 10:** Question 7. Please indicate how well each statement applies to you. (Please select one response for each item)

		Does not apply at all	Hardly applies	Rather does not apply	Neither applies nor does not apply	Rather applies	Mostly applies	Applies very much
1	I believe that choosing the nursing profession was the right decision for me.	1	2	3	4	5	6	7
2	I feel that I cannot imagine working in a profession other than nursing.	1	2	3	4	5	6	7
3	I intend to continue working in the nursing profession throughout my life.	1	2	3	4	5	6	7
4	I believe that becoming a nurse reflects who I am and the way I want to live.	1	2	3	4	5	6	7
5	I feel that I can say with pride to others that I am a student aspiring to become a nurse.	1	2	3	4	5	6	7
6	I have a clear idea of the kind of nursing care I want to provide.	1	2	3	4	5	6	7
7	I believe that I can practice nursing in a way that is true to myself.	1	2	3	4	5	6	7
8	I have a clear vision of the kind of nurse I want to become.	1	2	3	4	5	6	7
9	I believe that in the future I will be able to provide nursing care that reflects who I am.	1	2	3	4	5	6	7
10	I believe that I have my own perspective on the nature and role of nursing.	1	2	3	4	5	6	7
11	I believe that, as a nurse, I am an indispensable member of the healthcare field.	1	2	3	4	5	6	7
12	I believe that, as a nurse, I have been and will continue to be needed by many people.	1	2	3	4	5	6	7
13	I believe that, as a nurse, I am needed by patients.	1	2	3	4	5	6	7
14	I believe that, as a nurse, I will become increasingly necessary as a member of the healthcare team.	1	2	3	4	5	6	7
15	I believe that nursing is a profession grounded in a unique and well-established academic discipline.	1	2	3	4	5	6	7
16	I want to contribute to patients as a nurse.	1	2	3	4	5	6	7
17	I want to respond to and support patients’ wishes as a nurse.	1	2	3	4	5	6	7
18	I want to contribute to society as a nurse.	1	2	3	4	5	6	7
19	I want to contribute to the advancement of healthcare as a nurse.	1	2	3	4	5	6	7
20	I want to contribute to the development of the nursing profession as a nurse.	1	2	3	4	5	6	7

This concludes the questionnaire. Thank you very much for your cooperation.
